# Have LEGO Products Become More Violent?

**DOI:** 10.1371/journal.pone.0155401

**Published:** 2016-05-20

**Authors:** Christoph Bartneck, Qi Min Ser, Elena Moltchanova, James Smithies, Erin Harrington

**Affiliations:** 1 HIT Lab NZ, University of Canterbury, Christchurch, New Zealand; 2 Mathematics and Statistics, University of Canterbury, Christchurch, New Zealand; 3 School of Humanities and Creative Arts, University of Canterbury, Christchurch, New Zealand; University of Waterloo, CANADA

## Abstract

Although television, computer games and the Internet play an important role in the lives of children they still also play with physical toys, such as dolls, cars and LEGO bricks. The LEGO company has become the world’s largest toy manufacturer. Our study investigates if the LEGO company’s products have become more violent over time. First, we analyzed the frequency of weapon bricks in LEGO sets. Their use has significantly increased. Second, we empirically investigated the perceived violence in the LEGO product catalogs from the years 1978–2014. Our results show that the violence of the depicted products has increased significantly over time. The LEGO Company’s products are not as innocent as they used to be.

## Introduction

Violence has become an everyday occurrence in television, games and toys, and this is of particular concern when it comes to media aimed at children. Such violence has increased over time; for instance, [1] demonstrates that television programmes aimed at children contain more violence that other forms of programming, and [2] found a statistically significant increase in the duration of violence in G-rated English language childrens’ films released between 1937 and 1999.

### Effects of violence

Many psychologists have investigated the influence of media on the development of children. Previous studies have found that violent media and games are linked to negative behaviours [3–7], cognition, and affect [5, 8]. For instance, it has been demonstrated that frequent exposure to violent content results in desensitization (the elimination of emotional, cognitive, and behavioural responses to violence) [9], and is also linked to a decrease in empathy [6]. While this correlation between violence and negative social outcomes could be due to said desensitization, it could also be argued that people who are prone to violent behaviours are attracted to violent media [10]. Conversely, prosocial video games, in which a player must help other characters, have been demonstrated to have positive social outcomes [7].

That said, some studies contradict these findings; for instance, a recent study by [11] suggests that there is little to no correlation between violent media and aggressive behaviours. These contradictary study results could be due to ambiguous definitions or poorly designed research [11, 12]. So, while this is a much-studied area, these discrepancies suggest that we do not yet have a clear understanding of the effects of violent media content upon children’s development.

### Definition and measurement of violence

Although violence is widespread in contemporary society, researchers struggle to accurately define or measure it. Many broad definitions exist, such as that of the American Psychological Association (2015), which defines violence in terms of extreme aggression, citing examples such as assault, rape or murder [13]. Despite this, they do not provide a detailed coding scheme on how to quantify the intensity of violent acts.

Several coding schemes are available to assess the violent behaviour of children [14]. Such measurements are often used to investigate the relationship between exposure to violent media and aggressive behaviour [15]. Violence ratings for video games and movies, such those supplied by the US-based Entertainment Software Rating Board (ESRB), are usually decided by a panel of parents, who discuss the content of the material submitted before rating it [16, 17]; the same is the case for the ratings assigned to films in the United States by the Motion Picture Association of America (MPAA). Although this method avoids the need for a robust violence coding definition, it is less than useful as a protective mechanism due to increasing levels of violence in the wider media and entertainment industries, and the potential resultant desensitization of the raters themselves. For example, the iconic first person shooter “Doom”, which was released in 1993 and is considered by many to be the archetype of modern 3D shooting games, was put on the index by the Federal Department for Media Harmful to Young Persons in Germany, which meant it could not be advertised, and only sold to adults. However, in 2011 the game and its sequel were removed from the index based on a request from its makers. Cultural values had clearly shifted to the point where the game was no longer considered harmful, indicating an adjustment in the assessment of video game violence.

Many studies that conduct media and game violence correlational studies use a quantitative approach by simply counting the number of violent acts performed [18], although some entirely omit the method used to classify violence, possibly distinguishing violent material using their gut feelings or by using extreme cases of violence against non-violence [3, 4, 19].

Content analysis is often used to analyze the violence in television shows. In this widely-used method, the research defines a list of violent acts that the coders then count during the television show [20]. Content analysis thereby focuses on the quantity of violent acts, but is unable to make any statements about the intensity of the violence. While this makes the study much easier to conduct, it also results in a trade-off in validity. A study conducted by [21] found that the nature, degree, and amplitude of a particular violent act are just as important as the number of times it is committed, thus invalidating, or at least rendereding less helpful, most previous quantitative violence studies.

### Toy violence and child development

In an increasingly technological world, many violence studies are conducted in the field of media and video games. However, even children in the digital age are usually exposed not only to TV and video games, but to a mixture of digital and non-digital toys such as trains, Barbie dolls, and LEGO bricks.

Studies relating to the use of toy guns by children indicate how complicated the issues surrounding violent toys and childhood development are. In a study conducted by [22], it was found that toy guns correlated with a higher rate of antisocial behaviour in children as compared to nonviolent toys. This result backs up previous research findings by Carlsson-Paige and Levin, Frazier et al., and Silva (as cited in [8]) that violent toys are detrimental to children’s healthy development. Pediatricians also advise against buying toys that promote violence for children as the toys provided by parents represent important values [23]. On the other hand, toy guns are used in play therapy by professional therapists [24], as it was found that playful aggression is highly beneficial for child development [25]. One possible reason for the stark contrast in findings could be the distinct differences between serious aggressive behaviour and playful aggressive behaviour [25]. The intention to harm is found to be the major factor distinguishing serious aggression from playful aggression [25]. Supervision is a crucial component when allowing playful aggressive behaviour, as reinforcement should be given to ensure developmentally appropriate play [25].

Although the aforementioned studies assess the effects of toy violence in child development, there is little research studying how violent the toys themselves have become; that is, how the toys’ design might connote, encourage or depict violence, aggression or anti-social behaviour. This study is based on a supposition that media violence might influence the development of non-digital toys as well as digital toys like video games, resulting in greater amounts of violence in toys as a whole. By ignoring the complex relationships between digital and non-digital toys, researchers miss a crucial component in the imaginative lives of children.

This study aims to help fill this gap in the literature of toy violence by analysing LEGO products. The LEGO company has become the world’s largest toy company [26], and it produced more than 60 billion bricks in 2014 alone. LEGO products are sold in more than 140 countries, making it one of the most widely available toys in the world. LEGO bricks have even become a transmedia phenomenon [27] that has, at times, a cult-like status amongst its fans [28]. LEGO bricks, first patented in 1958, have also enjoyed a continuous production run, making them one of the few products that have been sold continuously for more than half a century. LEGO products therefore allow us to analyze the toy market from a historical perspective, complementing other studies including one that suggested the faces of LEGO Minifigures have become increasingly diverse and aggressive over time [29]. This study addresses gaps left by previous research in related areas. For instance, Oppel conducted a product analysis of toy guns and their use from a design perspective [30]. He discovered that an increasing number of LEGO and Playmobile sets have guns (see page 60) but it is not clear how the weapons were counted. The study only considered the sets from 1990–2013, thus limiting its scope.

**Fig 1:**
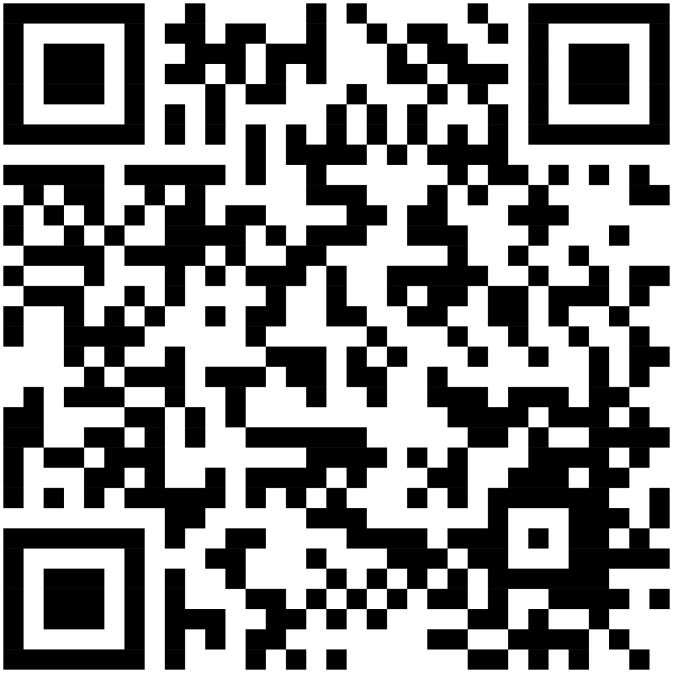
LEGO set 44001 Pyrox (Removed from this Manuscript due to licensing restrictions of PLOS One. Image available at: http://www.bartneck.de/publications/2016/legoViolence/44001.jpg.

This study explores a similar set of questions in relation to LEGO sets. The design of the core LEGO brick itself has remained largely unchanged, but the models released by LEGO, including their specialist pre-formed bricks and playsets, have changed and developed over time. Certain sets certainly seem to have increasingly aggressive themes, such as set 44001 “Pyrox” (see Fig 1), from LEGO’s Hero Factory range, which is described as a ‘battle-ready fire minotaur’. Products and play sets like these seem to have become more frequent in recent years, particularly in comparison to LEGO’s products from the 1970s.

### Research questions

In light of this anecdotal evidence, we were interested in exactly how violent LEGO products have become. To answer this question we conducted two studies. First, we investigated the weapon bricks produced over time. More specifically, we analyzed changes to the proportion of LEGO bricks that are weapons and the proportion of LEGO sets that contain weapons, along with the number of new weapon bricks introduced each year. Second, we investigated how the perceived violence of LEGO products has changed over time.

We would like to point out that our study does not aim to investigate the effects the depiction (direct, implied, or potential) of violence in LEGO products has on children. Although such a study would be desirable it presupposes that we have a clear understanding of how violent LEGO products are in the first place. Our study focuses on this first step: understanding how violent LEGO products are, and if this has changed over time.

## LEGO Weapon Bricks

It is reasonable to assume that the inclusion of weapon bricks, such as swords, guns, and cannons in LEGO sets indicates a degree of violence (direct, implied, or potential). That said, there are two limitations to basing a method on this assumption. First, not every weapon brick in a set may be used in the model for this purpose. Second, there are bricks that are not designed to be specifically used as weapons, but that may be used as such in the model. Moreover, multiple non-weapon bricks can be assembled to represent a weapon. The Star Wars Death Star, for example, is certainly a weapon, but it consists of many non-weapon bricks. By our definition, the Death Star itself would not be considered a weapon brick.

We used the LEGO set inventory lists from BrickLink.com as the basis for this analysis. BrickLink.com is the world’s largest online marketplace for after-market LEGO trading. Their database of LEGO parts and sets, though imperfect, is the most complete and up-to-date data source that is publicly available. We used BrickLink.com’s part category of “Minifig, Weapon” as the definition of a weapon brick. We checked all 155 parts in this category and all of them were weapon or weapon related. Although imperfect, this seems to be the best approximation for a definition for a weapon brick. Our definition of weapon brick is likely to underestimate the available weapons since it does not include all weapons, such as those available in the highly weaponized Bionicle theme.

### Data Processing

The data from BrickLink.com contains the exact inventory of every brick in every set the LEGO company released since 1949. The data for the year 2015 is still incomplete since not all sets scheduled for this year have yet been released. It is also important to note that the BrickLink.com database records only the release of a set and not the period over which the set was sold. The Death Star (set 10188) was released in 2008 and is recorded as such in the BrickLink.com database. However, it has been sold continuously since then and is possibly the longest selling set so far, given that most sets are not typically sold longer than two years. Another important consideration is that our analysis does distinguish between shapes and colors of individual weapon bricks. Although the BrickLink.com database only gives a single identification number to a part, we consider a brick in different colors to be a different weapon brick. A brown tube possibly represents a stick while a grey tube of the exact same design could represent a metal rod. While an attack with both would be painful, a metal rod could certainly be interpreted as more dangerous.

### Measurements

The number of sets released per year and the number of bricks in every set varies considerably across time. The number of sets released each year, for example, has increased dramatically. In the year 1970, 35 different sets were released. This number grew to 419 sets in 2010. It is therefore necessary to count not only the number of sets that contain weapon bricks, but to contextualise this number with regards to the total number of sets released in that year.

The same holds true for the raw number of weapon bricks per year, which have to be considered in proportion to the total number of bricks of that year. The latter is defined as all the bricks in all the sets of a given year, including duplicates. A certain set might contain three times the same brick. This will add a count of three to the total number of bricks of that year. We define the measurements as:

- The *count of weapons* is the count of new weapon bricks designs released per year. This measure does not take colour variations into account.
- The *proportion of weaponed sets* is the count of all sets that contain at least one weapon brick in a given year divided by the total number of sets in that year.
- The *proportion of weapon bricks* is the count of all weapon bricks in all sets of a given year divided by the total count of all bricks in all sets of that year.

### Results

A generalized linear model (GLM) from a Poisson family was fitted to analyze the possible temporal trend in the count of weapons (M1), and a linear model was fitted to analyse the possible temporal trend in the proportion of weapon bricks (M2) and the proportion of weapon sets (M3) released in each respective year. Due to the nature of the variable and the need to satisfy the linear model assumptions, the *logit* transformation was used on the response, and the interpretation of the trends for the models M2 and M3 are thus in terms of odds rather than proportions. Year of release was the only covariate in the model. The models were fitted within a Bayesian paradigm [31] using WinBUGS software [32] and R software [33], including the package R2WinBUGS [34]. Non-informative Gaussian priors with mean 0 and variance 100^2^ were used for the regression coefficients, 10000 iterations were run after a 5000 iterations’ burn-in and the convergence was assessed by visual inspection. The model details and the trend estimated and credible intervals (CI) are shown in Table 1.

**Table 1:** Estimated trends in the count of weapons, proportion of weapon bricks and sets in LEGO by year of release.

	Response Variable *Y*	Model Likelihood	Posterior mean estimate for (*exp*(*b*) − 1)*100%	95% CI
M1	weapon count	*Y* ∼ Poisson(exp(a + bt))	10.8	(8.5;13.5)
M2	proportion of w. bricks	$\text{log}(\frac{Y}{1-Y}∼N\left(a+bt,\tau \right)$	5.7	(3.1–8.4)
M3	proportion of w.sets	$\text{log}(\frac{Y}{1-Y}∼N\left(a+bt,\tau \right)$	7.6	(5.6–10.1)

For the weapons count, a strong positive annual trend of an average 10.8% was found (95% CI: 8.5–13.5%).

**Fig 2:**
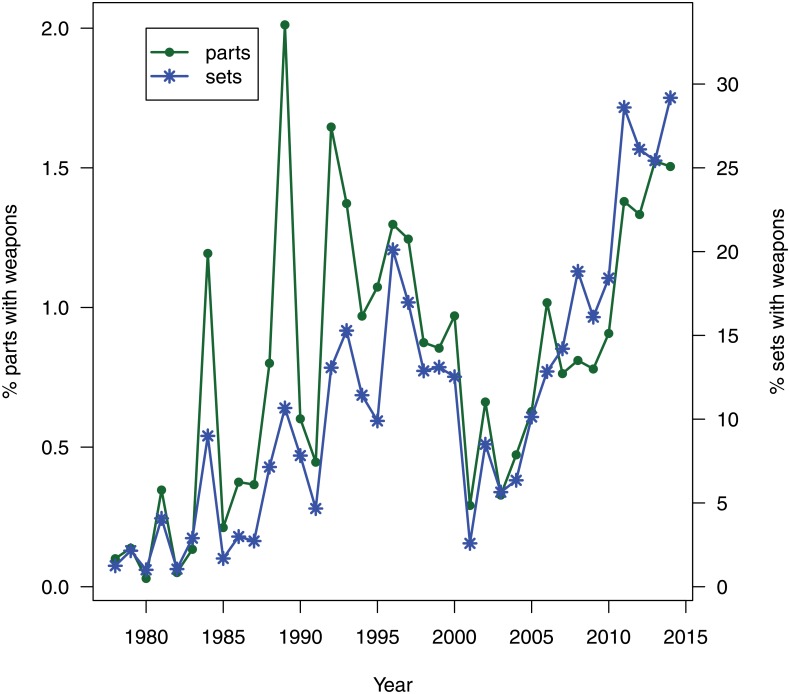
Weaponed sets across time.

Next we investigated the temporal dynamics of the proportion of weapon bricks and weapon sets. The two were strongly correlated (*r*^2^ = 0.78) as shown in Fig 2. The odds of a brick or a set having weapons increased annually by an average of 5.7% (95% CI: 3.1–8.4%) and 7.6%(95% CI: 5.6–10.1%) respectively. However, the dynamic is not monotonic: the proportion of weaponed sets increased steadily for the first twenty years and dropped below 5% in 2001 before climbing back up to nearly 30% in 2014 (Fig 2).

### Discussion

**Fig 3:**
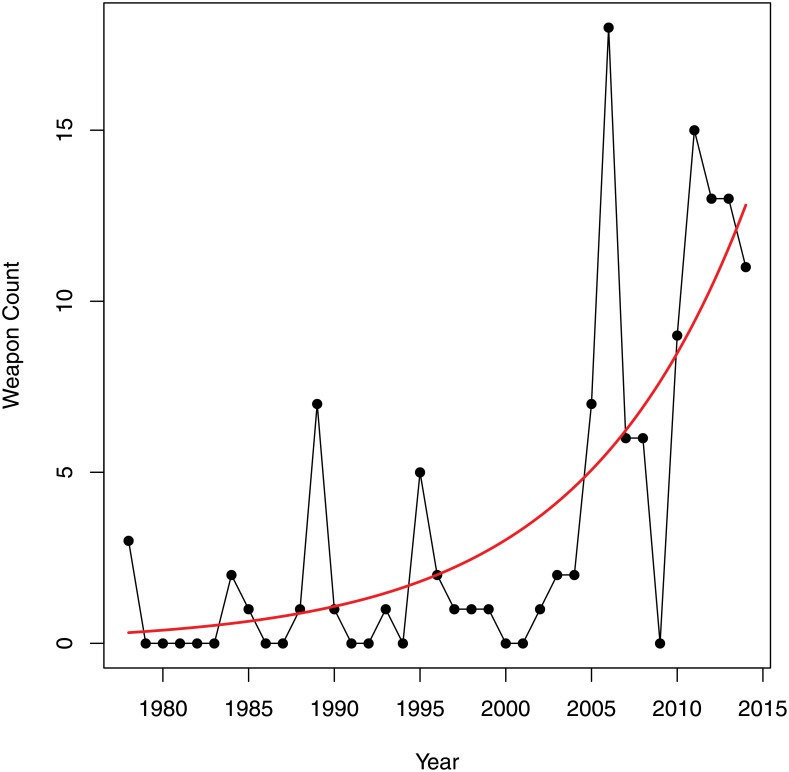
Number of weapons over time.

The first weapon bricks—a sword, a halberd, and a lance—were released in the year 1978. We will therefore focus our analysis on the years 1978–2014. Fig 3 shows the count of weapons across time.

Weapon bricks are largely associated with themed LEGO sets. The first weapon bricks, described above, were part of the Castle theme. In 1989 the Pirates theme, which included handguns and cannons, was introduced. This also resulted in a spike in the proportion of weapon bricks. 1995 showed another spike. This is associated with the introduction of the Aquazone themes, which contain harpoons and knives. In the years 2005 and 2006, many new weapon bricks associated to the Bionicle theme were released.

While the release of the Star Wars theme in 1999 only introduced one new weapon brick, the light saber, more weapons followed in 2007 in the shape of rifles and blasters. The Star Wars theme started a trend of licensed LEGO franchise products [35], but this did not appear to cause many changes in the trends of proportion of weapon bricks or proportion of weaponed sets. The increase in violence due to the Star Wars theme might have initially been offset by the discontinuation of several highly-weaponed themes such as Insectoids, UFO, and Rock Raiders [35]. The launch of the themes based on the Lord of the Rings films sparked a new series of weapon bricks in 2012 and the following years.

There has been a significant increase in the proportion of weaponed sets and the proportion of weapon bricks across time. Today nearly 30% of LEGO sets contain at least one weapon brick, and this number does not even include weapons that consist of an assembly of non-weapon bricks.

The year 2001 seems to be a turning point in several respects. There were no new weapon bricks introduced and the proportion of weaponed sets fell below five percent. This dip could be related to the financial problems the LEGO Group encountered, which resulted in the retrenchment of an estimated 1000 employees [36]. These dramatic changes might have led to a stagnation in the number of new bricks and sets being created. It may have also relatde to the revision in the company’s mission to be more children-oriented [37], which might have resulted in the release of more children-friendly.

## Perceived violence of LEGO products

**Fig 4:**
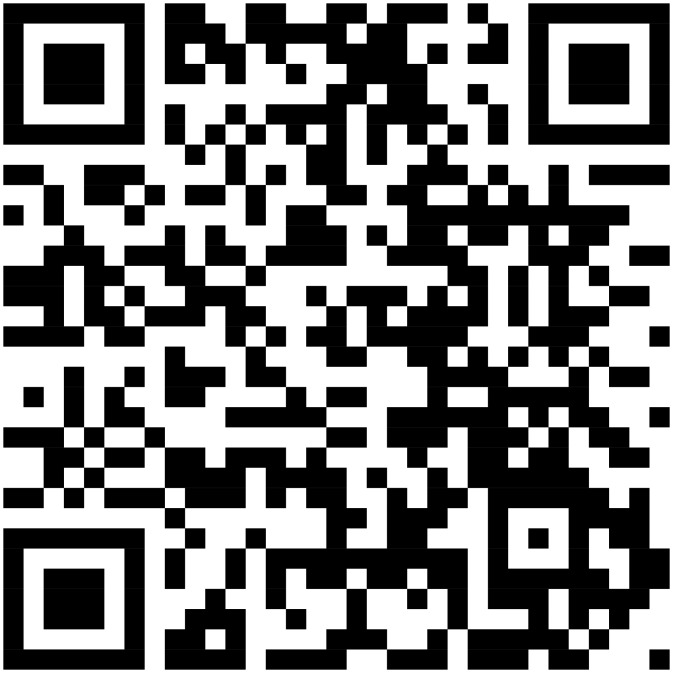
A page from the 2014 LEGO catalog (Removed from this Manuscript due to licensing restrictions of PLOS One. Image available at: http://www.bartneck.de/publications/2016/legoViolence/legoCatalogPage.png.

The LEGO company has released more than 12,000 sets since 1949. It seems unpractical to attempt to analyze all these sets individually. Instead we decided to use the LEGO product catalog that the company produced once or twice a year since 1950. These catalogs do not only show most of the LEGO sets, but they also put them into the context of a play scenario (see Fig 4). The Minifigures and models act out their intended behaviors in these scenes. The LEGO catalogs are therefore an ideal data set through which to analyze the violence in LEGO products.

### Measurements

We were unable to identify a suitable measurement for assessing the violence in still images. We therefore adapted the violence coding scheme from the study conducted by [21]. As the coding scheme was originally designed to rate television violence, we simplified it to fit our study by removing aspects of the coding scheme that were irrelevant to the rating of still images. For example, the mode of verbal aggression and the duration of the act were impossible to code for still images. The section for coding attractiveness of violence was also removed as it was irrelevant to our study.

The simplified violence coding scheme contained eight questions:

1. mode(s) of physical violence
2. mode(s) of nonverbal psychological aggression
3. atmosphere of violent act(s)
4. realization of violence
5. consequences of violence
6. temporal distance
7. clarity and vividness of violent act(s)
8. intensity of violence (a summative rating)

The answers for the first three questions were nominal and in the form of check boxes. Participants could choose multiple answers. The responses for questions four to eight were ordinal and in the form of radio buttons where only one answer could be selected. Questions three to eight only became available if the participants selected at least one type of physical or psychological violence. The questionnaire also included demographic questions such as gender, age, and ethnicity.

### Setup

We used the Crowdflower platform to recruit and execute our study. Results acquired from crowdsourcing platforms such as Crowdflower and Amazon Mechanical Turk are similar in quality to results that are obtained from traditional methods [38].

We conducted a pilot study in which we asked twenty images to be rated by 20 participants. We then performed a power analysis to estimate how many participants would be required to detect an increase in violence perception with the probability of at least 80%. We have estimated that at least 15 images per decade with at least three raters per image would be sufficient, and thus we set up the experiment so that each image was rated by at least three participants.

Participants could rate as many images as they desired but they had to spend at least 20 seconds rating each image. Participants that answered more quickly than this were banned from further providing any answers. This procedure ensured the quality of the responses obtained.

### Stimuli

We were only able to acquire the LEGO catalogs from the year 1973 forward in a sufficient quality. The catalogs were processed into one image per page. Several pages were excluded since they contained content that did not show any actual LEGO products, such as advertisements for the LEGOLAND theme parks. In addition, we excluded pages that did not use the standard LEGO scale, such as those featuring DUPLO or Junior products. The remaining 1576 images were resized proportionally to a maximum width of 768 pixels.

**Fig 5:**
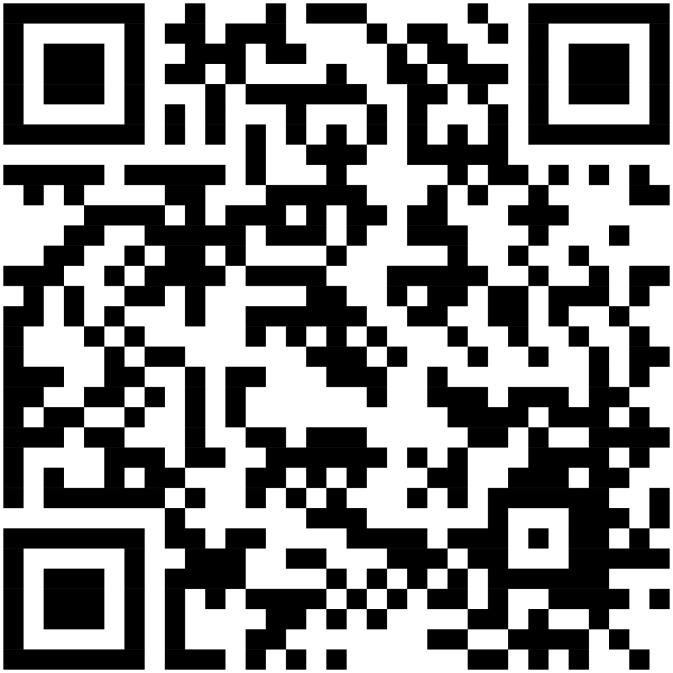
Screenshot of example task (Removed from this Manuscript due to licensing restrictions of PLOS One. Image available at: http://www.bartneck.de/publications/2016/legoViolence/task.png.

According to Browser Display Statistics from W3Schools (2015) [39], 88.7% of visitors to their site had a screen resolution of 1280x1024 pixels or higher. Therefore the study was designed to show participants images with a maximum width of 573 pixels, to ensure that the majority of participants could see the image in its entirety whilst answering the questions side-by-side, as seen in Fig 5 below. Participants could view the full-sized image with a width of 768 pixels in a separate window when they clicked on the image. The order of the catalog images was randomized.

### Participants

The participants were recruited using CrowdFlower on 19 May 2015. Of the 161 participants who took part in the study, 70.2% were males, 25.5% were females, and the rest had missing gender data. The mean age of the participants was 31.2 with a standard deviation of 9.01 in a range of 18 to 54 years old. The workers were recruited to participate willingly in the current study using a variety of channels on CrowdFlower, and were paid a rate of $6 per hour. The participants in the experiment had the opportunity to enter a contributor satisfaction rating for our experiment within the Crowdflower system. The average rating was 4.4 our of 5, which indicates that the contributors felt the amount of workload and pay was reasonable as compared to other jobs available in CrowdFlower.

### Results

A total of 4728 responses from 161 workers were collected, resulting in the average of 29.3 responses per person. Participants spent an average of 37 seconds on each page. Of those 152 have entered valid age and gender, shown in Table 2, and 148 have entered valid ethnicity data as follows: 33 Caucasian, 30 Latino/Hispanic, 6 Middle Eastern, 45 South Asian, 18 East Asian and 16 Other.

**Table 2:** Demographics of the LEGO violence perception Survey: Sex and Age.

Age:	Male	Female
18–24	28	9
25–34	54	17
35–44	18	7
45–54	11	8

Out of 4728 total responses, 359 were deleted due to the overly low trust score (< 0.70), as were a further 11 due to technical problems during the survey. The trust score is a parameter provided by Crowdflower and is based on the responses participants make to quality control questions in all the jobs they accept. We excluded participants who have a trust score lower than 70%.

Responses to each of the survey questions were checked for consistency. If a participant responded to question two ‘Mode of nonverbal physical aggression’ with both ‘none’ and ‘threatening, intimidation’, then this response was deemed inconsistent and omitted from further analysis. Note, that the questions 4 to 8 were only answered if the image was perceived as violent as defined by the participants answer to question 1 or 2.

Since the answer options for within questions 1–3 were not mutually exclusive, each answer option was analyzed separately using logistic regression. Questions 4–8, on the other hand, had mutually exclusive answer options that increased in intensity and were therefore analyzed using ordinal cumulative logistic regression with proportional odds [40]. All the models were adjusted for sex, age group and ethnicity, although none of those factors was found to have had a significant effect on the findings. Perhaps somewhat surprisingly, people in the youngest age-group, 18–24 were found to perceive violence more often than 25–44 year olds, but less often than 45–54 year olds.

Repeated measures were taken into account via random effect terms for individual participants and individual catalogue pages. The models were fitted within a Bayesian paradigm [31] using WinBUGS software [32] and R software [33], including the package R2WinBUGS [34]. All the random and fixed effects were given non-informative priors. The technical details of the ordinal multinomial logistic regression model can be found in the WinBUGS code supplied in the Appendix B. model A total of 5000 iterations were monitored after a 5000 burn-in. Convergence was confirmed by visual inspection. The technical specifications of the model and the WinBUGS code use in estimation can be found in Appendices B and C respectively.

**Fig 6:**
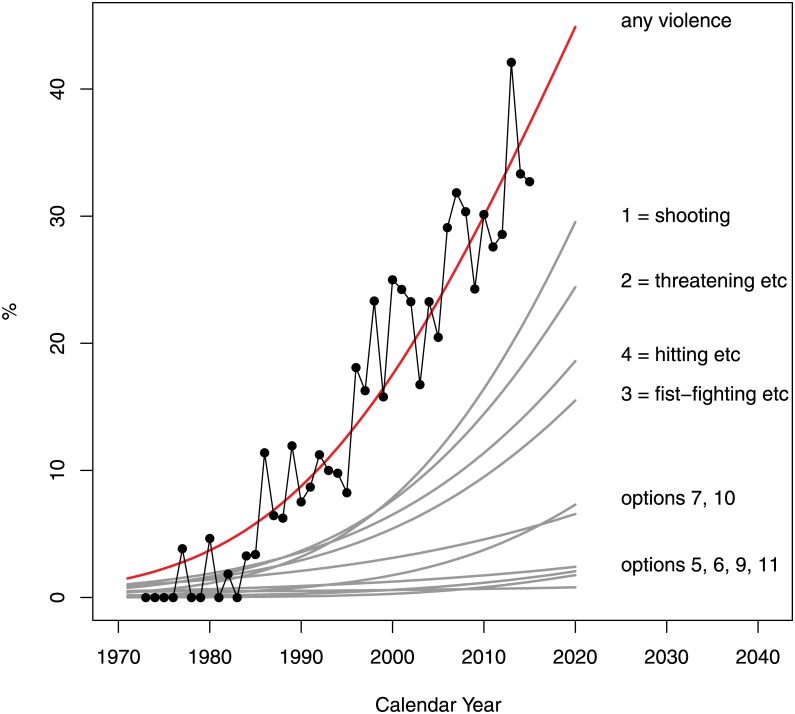
Rate of increase in the mode of perceived physical violence (Question 1 of the Survey). The options were as follows: 0 = none, 1 = shooting, 2 = threatening or forcing with guns, 3 = fist-fighting, pushing, striking, 4 = hitting with weapons/tools/knockout, 5 = strangling, 6 = poisoning, 7 = slashing, 8 = sexual violence, 9 = kidnapping/ tying up/ arresting, 10 = damaging property, 11 = other.

The odds of presence of any type of physical violence (question 1) were perceived to increase significantly at an average rate of 19% per year (95% CI: 16–23%); see Fig 6. By 2010–2015, about 40% of the catalog images were perceived as containing some kind of violence. The fastest growth has occurred in cases of shooting (estimated average 17% per year with 95% CI: 14–21%) and the slowest in cases of hitting with weapons / tools / knockouts (estimated average 7% per year with 95% CI: 6–9%). No cases of perceived sexual violence were reported.

**Fig 7:**
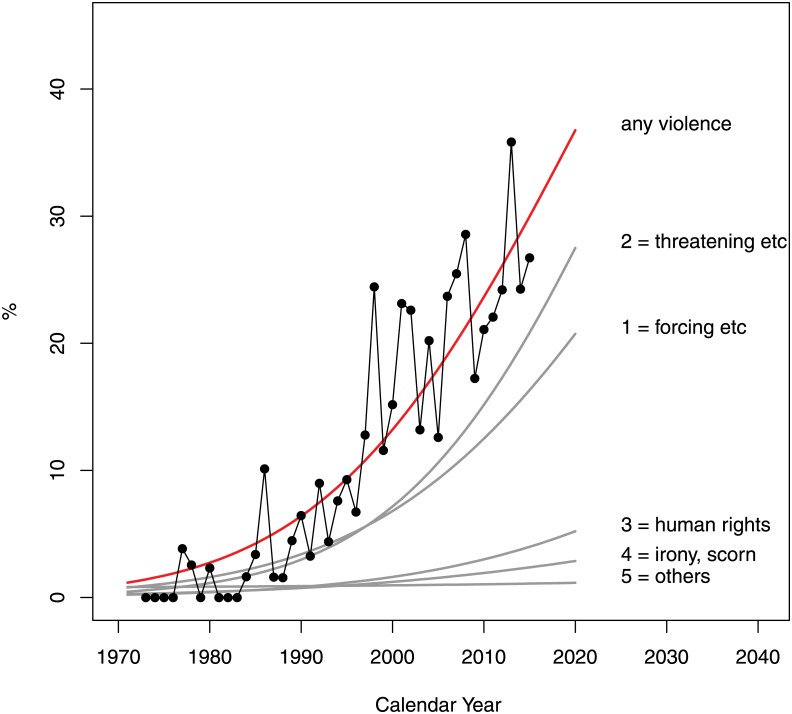
Mode of nonverbal psychological aggression (Question 2 of the Survey). The options were as follows: 1 = forcing, subjection, pressuring 2 = threatening, intimidation 3 = violating one’s human rights 4 = irony, scorning gestures 5 = other.

The odds of the presence of nonverbal psychological aggression (question 2)—classified as forcing, subjection, pressuring (1), threatening, intimidation (2), violating one’s human rights (3), and irony, scorning gestures(4)—were estimated to grow significantly at 11.7%, 16.1%, 14.2% and 8.4% respectively with the 95% CI: 9.0–14.8%, 12.9–19.9%, 7.2–26.9%, 3.5–15.1% respectively (see Fig 7).

**Fig 8:**
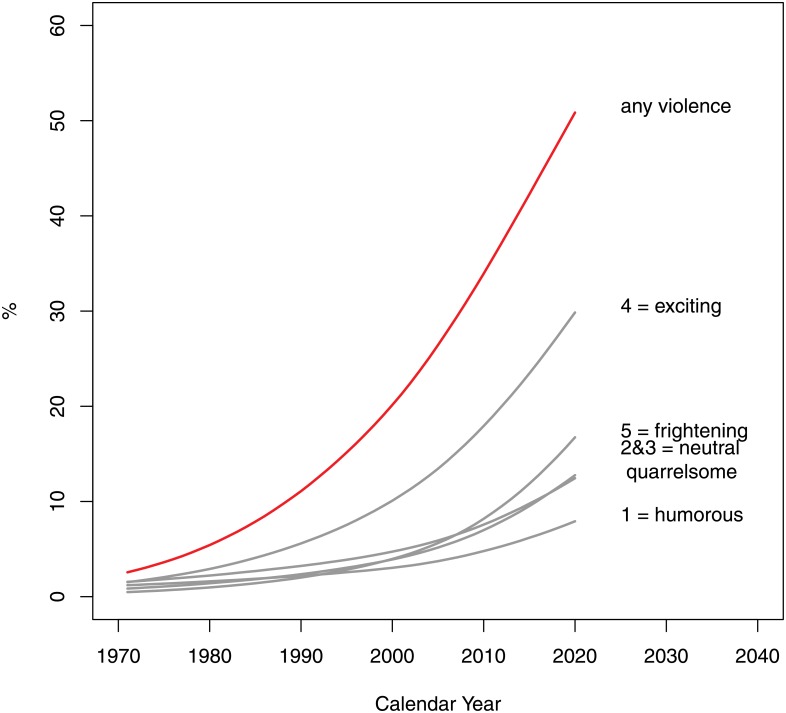
Dramatization: atmosphere of violent act(s) (Question 3 of the Survey). The options were as follows: 1 = humorous, comic; 2 = neutral or unclear; 3 = quarrelsome; 4 = exciting, adventurous; 5 = frightening, threatening, horrific.

The odds of presence of violent atmospheres (question 3) perceived as humorous or comic (1), neutral or unclear (2), and quarrelsome (3) all increased significantly at approximately the same rates of 7.8% (95% CI: 6.8–10.8%). The increase in the odds of exciting and frightening situations was estimated at 11.8% and 13.1% respectively with 95% CI: 11.0–14.4% and 11.6–17.5% (see Fig 8).

**Fig 9:**
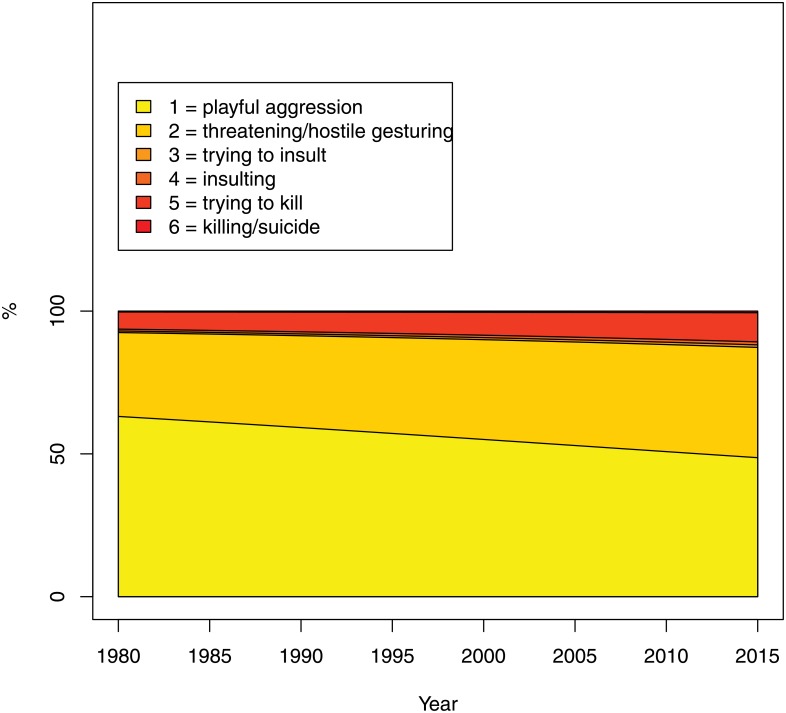
Seriousness: realization of violence (Question 4 of the Survey). The percentage on the Y-axis refers to the estimated probability of falling into each category.

The level of seriousness of the realization of violence (question 4) was perceived to increase slightly: while in 1980 an estimated 63% of all violence were perceived as playful, as opposed to threatening and trying to kill, in 2015 this proportion came down to 49% (see Fig 9). The trend, however, was not statistically different from zero.

**Fig 10:**
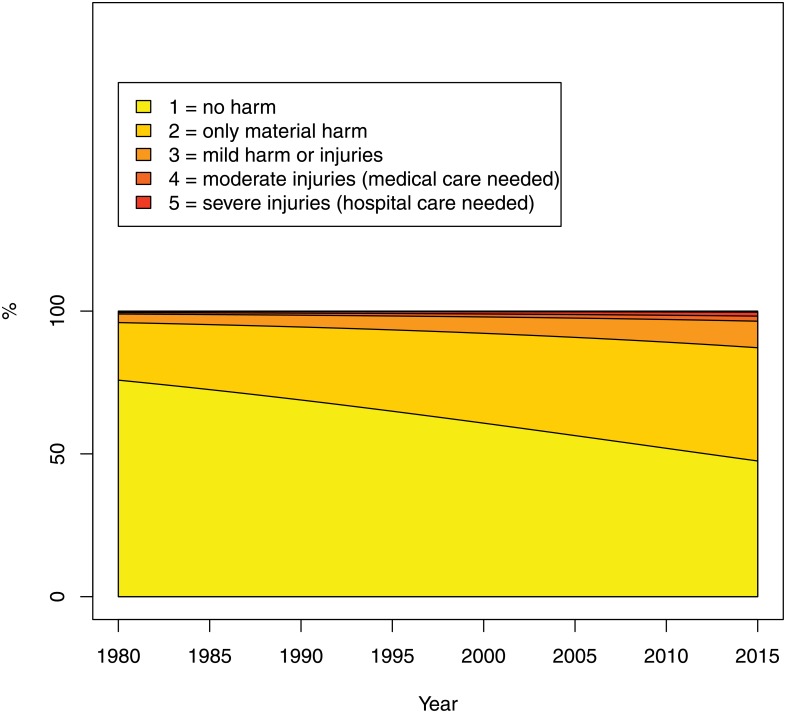
Seriousness: the consequences of violence (Question 5 of the Survey). The percentage on the Y-axis refers to the estimated probability of falling into each category.

The seriousness of the portrayed consequences of violence (question 5), on the other hand, has significantly increased: the odds of harmful consequences as opposed to none at all were estimated to increase by an average 2.7% per year (95% CI: 1.3–4.4%) (see Fig 10).

**Fig 11:**
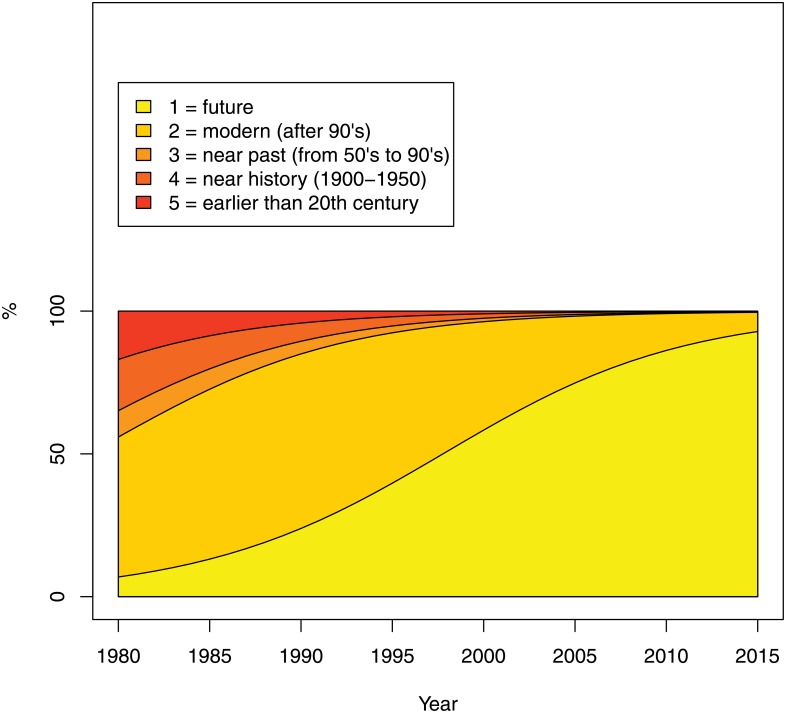
Realism: temporal distance (Question 6 of the Survey). The percentage on the Y-axis refers to the estimated probability of falling into each category.

The realism of the violence in terms of its temporal distance (question 6) changed significantly over time: the odds of the violent scene being placed further into the future increased by an average 17% per year (95% CI: 15–23%). In 1980 around 10% of the catalog pages were estimated to be set in the future, while by 2015 that proportion climbed to an estimated 90% (see Fig 11).

**Fig 12:**
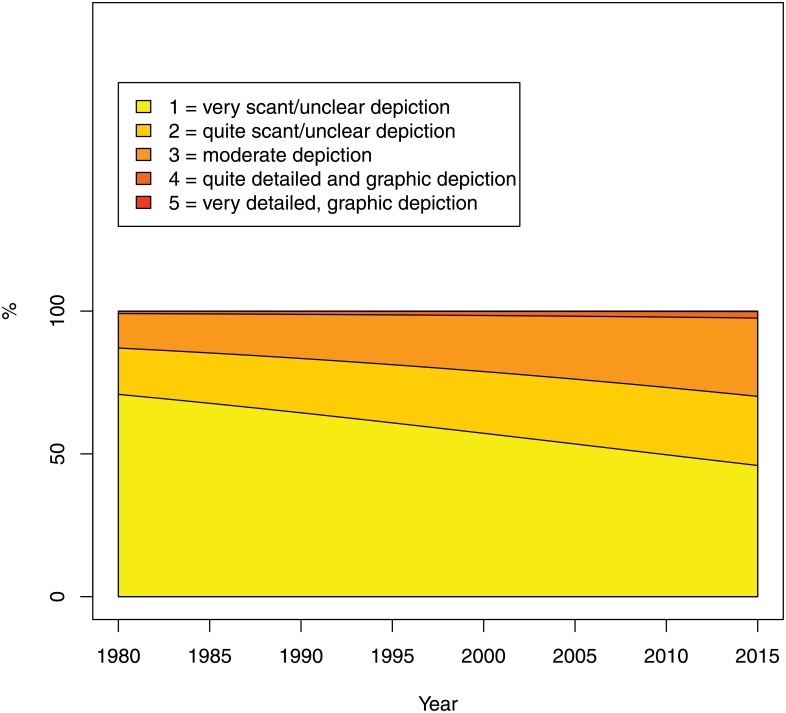
Dramatization: clarity and vividness of violent act(s) (Question 7 of the Survey). The percentage on the Y-axis refers to the estimated probability of falling into each category.

The clarity and vividness of the dramatization of violent acts has also increased significantly, with the odds of falling into a more violent category increasing by an average of 3.0% a year, 95% CI: 0.9–5.1%), (see Fig 12).

**Fig 13:**
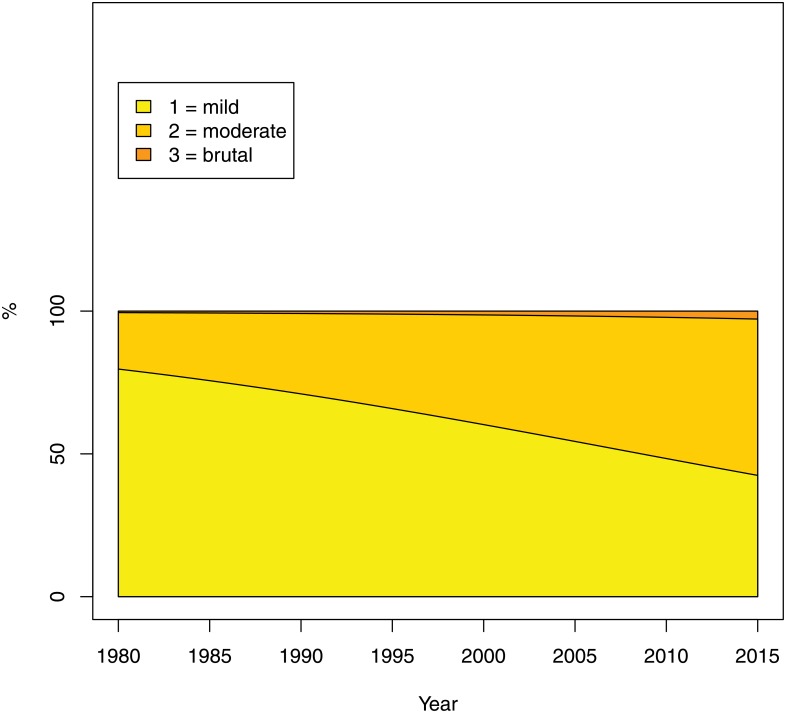
Intensity of violence (Question 8 of the Survey. The percentage on the Y-axis refers to the estimated probability of falling into each category.

The brutality of the portrayed violence was perceived to have increased significantly: while in 1980 only an estimated 20% were perceived to be moderately or brutally violent, in 2015 the corresponding proportion was estimated at 58%, (see Fig 13). Finally, there was also a clear move towards futuristic scenes (see Fig 11).

### Discussion

The perceived violence in LEGO products has increased significantly over the years. The chances of observing violence in a LEGO catalog pages has increased steadily by 19% per year. Currently, around 40% of all pages were coded by participants as indicating some type of violence. In particular, scenarios involving shooting and threatening behaviour have increased over the years. The perception of nonverbal psychological aggression increased at a similar rate. The atmosphere of the violent acts is predominately perceived as exciting. The LEGO company often claimed that their violence normally happens within a humorous context, yet the results show that “humorous” is the least likely atmosphere. Material harm is the most frequent consequence of the violent acts followed by mild harm or injuries.

## Conclusions

The results from both studies, weapons count and perceived violence, showed significant exponential increases of violence over time. LEGO products have become significantly more violent. This increase is not in line with their policy that “LEGO products aim to discourage pretend violence as a primary play incentive. The designs are meant to enrich play with engaging conflict scenarios where aggression might be used for the purpose of overcoming imaginary evil” [41]. The violence in LEGO products seems to have gone beyond just enriching game play.

It is unlikely that the LEGO company is the only toy manufacturer whose products have become increasingly violent; for instance, Oppel has already provided initial evidence that Playmobile has followed a similar trajectory. Within the spectrum of available products today, LEGO sets might still be comparatively or relatively harmless. The question remains, though, why violence has increased so much in general.

According to Smith and Zuiker (as cited in [42]), creators and producers of games and movies strive to push the limits of what violent media is allowed to be released to prevent their audience from getting bored of similar content. This creates content that is increasingly creative and violent. To catch the attention of their customers, toy manufacturers are similarly locked in a metaphorical arms race for exciting new products. In this race they do not only compete with other toy manufacturers but also with television and video games, which have also become more violent over the years.

It is unclear how this battle for attention can be moderated. Most countries do have policies and government agencies in place to protect children from excessively violent toys and media. Only Afghanistan, of all countries, has recently banned all toy guns in an attempt to curb a culture of violence [43]. It remains to be seen if they will be able to implement and enforce this law.

To provide effective protection from the effects of violence, government agencies need tools to assess the violence of toys and media. As indicateed earlier, most tools available for evaluating television programs are based on a count of violent acts without paying much attention to the nature and intensity of the violent acts. The situation for video games is even more ambiguous. While both movies and video games are subject to the monitoring of independent agencies, the exact method for their assessment remains informal. For this study we adapted a questionnaire initially used for television for still images. To our knowledge, there is no other tool available for this purpose. We hope that our questionnaire might be of use to other researchers when assessing the nature and intensity of violence in still images.

## Limitations and future studies

The definition of “weapon brick” does not necessarily include all possible weapons as it does not cover neutral bricks that could be built into weapons. As LEGO toys are bricks, and the nature of the toy is for smaller bricks to be built into something larger, studies on LEGO violence becomes more complex. In most cases of LEGO play, especially in larger weapons like cannons, slingshots, and Death Stars, many neutral bricks must be assembled into a weapon before it can be played with. Books such as “Forbidden LEGO” teach players how to build violent models using LEGO bricks [44]. With the increase in availability and popularity of such books, weapons built from neutral bricks would perhaps increase as well. On the other hand, bricks that were designed to be weapons but were not used as weapons during play were also classified as a weapon in this study. As there are many ways to view and use a brick, some players might not see a weapon brick as a weapon, but instead use it as a neutral brick or decoration. For example, though a sword is designed by LEGO to be a weapon, it may be used by some players as a decoration for a LEGO house. It is impossible to account for the specific use of bricks, by either adult collectors or children, although it is reasonable to assert that the images provided with themed sets offer suggested play scenarios.

**Fig 14:**
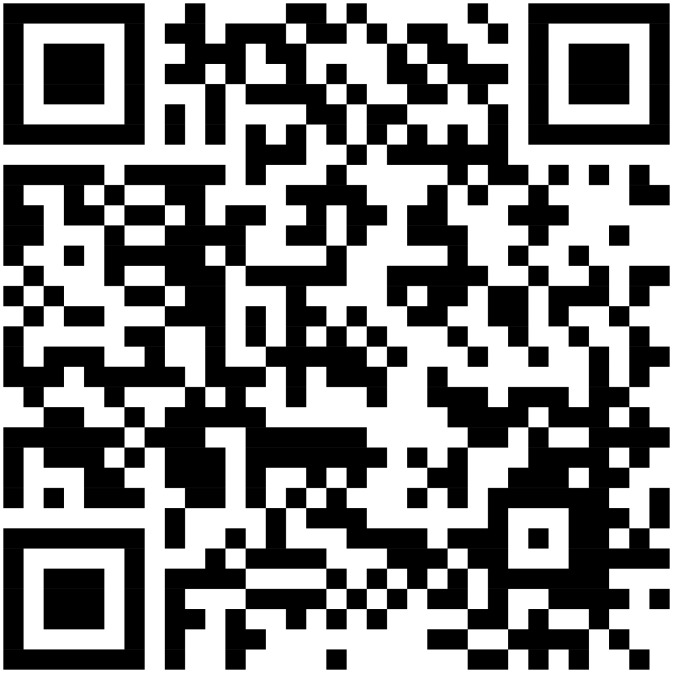
Call of Duty set by Megablocks (Removed from this Manuscript due to licensing restrictions of PLOS One. Image available at: http://www.bartneck.de/publications/2016/legoViolence/callOfDuty.png.

Future studies could also conduct a comparison between different toys. The LEGO products are certainly not the only toys that have changed over time. Two comparisons could be of particular interest. First, a comparison to Playmobile, another long standing product line, would allow for a historical review of the changes in their respective products. Second, a comparison to other construction brick systems could reveal how the LEGO products developed within this specific toy market segment. The Megablocks toys, which are completely compatible to the LEGO brick system, would be a good starting point. Megablocks is currently offering sets that are linked to violent computer games and film franchises such as Terminator, Call of Duty, Halo and Assassin’s Creed. In particular the Call of Duty product line is clearly based on modern military (see Fig 14), whereas the LEGO company has not yet released a battle tank. Compared to other toys, the LEGO products could still be considered to be relatively mild. Mads Nipper, the LEGO Group’s Senior Vice President in Global Innovation in Marketing even declared that “We will never produce realistic toys for playing war.” [41]

There is also a definite difference between perceived violence in images and perceived violence in the real world [45]. The images from LEGO catalogs may create an impression of comedy, which may have resulted in lowered violence ratings. Moreover, intentions are easier to be measured in reality as compared to images. Future studies could measure the intentions and violence levels in children playing with LEGO through behaviour observation.

Towards the end of our study we noticed a small inconsistency in the questionnaire. In question 6 “Temporal Distance” there is no option for the 19th century (1801–1900). Option five covers the range of 1900–1950 and option six is earlier than 1801. This inconsistency is probably based on the misinterpretation of the definition of the 19th century. This inconsistency occurs in the original questionnaire. Only very few sets fall into this category and hence we believe that it has no major impact on the results. Future studies should describe option six as “prior to 1900” to prevent any confusion.

## Appendix A: Simplified Coding Scheme for Lego Catalogue Violence

1. Mode of physical violence (Choose ALL mode(s) of physical violence that can be found):
  - none
  - shooting
  - threatening or forcing with guns
  - fist-fighting, pushing, striking
  - hitting with weapons/tools/knockout
  - strangling
  - poisoning
  - slashing
  - sexual violence
  - kidnapping/ tying up/ arresting
  - damaging property
  - other
2. Mode of nonverbal psychological aggression (Choose ALL mode(s) of psychological violence that can be found.)
  - none
  - forcing, subjection, pressuring
  - threatening, intimidation
  - violating one’s human rights
  - irony, scorning gestures
  - other
3. Dramatization: atmosphere of violent act(s) (Choose ALL atmosphere(s) of violence that can be found.)
  - humorous, comic
  - neutral or unclear
  - quarrelsome
  - exciting, adventurous
  - frightening, threatening, horrific
4. Seriousness: realization of violence (Choose the HIGHEST option that can be found in the image)
  - cannot code
  - playful aggression
  - threatening/hostile gesturing
  - trying to insult
  - insulting
  - trying to kill
  - killing/suicide
5. Seriousness: the consequences of violence (Choose the HIGHEST option that can be found in the image.)
  - portrayed not at all
  - no harm
  - only material harm
  - mild harm or injuries
  - moderate injuries (medical care needed)
  - severe injuries (hospital care needed)
  - death
6. Realism: temporal distance (Choose the HIGHEST option that can be found in the image.)
  - cannot code
  - future
  - modern (after 90’s)
  - near past (from 50’s to 90’s)
  - near history (1900–1950)
  - earlier than 19th century
7. Dramatization: clarity and vividness of violent act(s) (Choose the HIGHEST option that can be found in the image).
  - cannot code
  - very scant/unclear depiction
  - quite scant/unclear depiction
  - moderate depiction
  - quite detailed and graphic depiction
  - very detailed, graphic depiction
8. Intensity of violence (Choose the HIGHEST option that can be found in the image).
  - mild
  - moderate
  - brutal

## Appendix B: Bayesian ordinal multinomial logistic regression with random effects

Let *Y*_*i*_ denote an ordinal categorical response of a person *i* to a question about set *j* on a scale from 1 to *K*. For example, in Question 8 of our LEGO violence questionnaire, the intensity of violence could be graded as mild, moderate, or brutal. We then assume that the cumulative probability of *Y*_*i*_ taking on a particular value is structured as following

$$\text{logit}\left(Pr\left(Y_{ij}\leq k\right)\right)=\alpha_{k}+\beta *year_{j}+X_{i}*\gamma +\eta_{i}+\zeta_{j}$$

for *k* = 1, …, *K* − 1 where *α*_1_ < *α*_2_ < … < *α*_*k* − 1_; *β* is the effect of the year of the set release *year*_*j*_; *γ* is the vector of effects of respondent-specific covariates, such as age, sex and ethnicity, encoded in matrix *X*_*i*_; and *η*_*i*_ and *ζ*_*j*_ are person- and set-specific random effects respectively.

the coefficient *β* was assigned a Gaussian prior with mean 0 and variance 10^3^ whereas the elements of the vector *gamma* were assigned Gaussian priors with means 0 and variances 10^2^. The priors for the random effects were:

$$\eta_{i}∼\;N\left(0,\tau_{\eta }\right)$$

and

$$\zeta_{j}∼N\left(0,\tau_{\zeta }\right)$$

with the precision (inverse variance) parameters *τ*_*η*_ and *τ*_*ζ*_ being assigned Gamma priors Gamma(0.01, 0.01).

Finally, for the regression intercepts, the following priors were used:

$$\alpha_{1}∼U\left(0,20\right)$$

and

$$\alpha_{j+1}-\alpha j∼U\left(0,20\right)$$

for *j* = 1, …, *k* − 2.

Sensitivity analysis confirmed that the priors were non-informative.

Binary logistic regression can be considered within the same framework as a special case of the above model when *K* = 2. More about modeling of ordinal categorical data in general and ordinal multinomial logistic regression in particular may be found in [40].

## Appendix C: WinBUGS code for ordinal multinomial logistic regression with random effects

 # ORDINAL LOGISTIC REGRESSION

model;

{

# likelihood using categorical distribution

# Y [i] takes discrete values between 1 and K corresponding to ordinal multinomial categories

for(i in 1:N){

Y [i] ~ dcat(p [i,1:K])

}

# categorical probabilities obtained from cumulative probabilities

for(i in 1:N){

p [i,1] <- p.cum [i,1]

p [i,K] <- 1-p.cum [i,K-1]

for(k in 2:(K-1)){ # categories

p [i,k] <- p.cum [i,k]-p.cum [i,k-1]

}}

# cumulative probabilities

for(i in 1:N){

for(k in 1:(K-1)){ # categories

p.cum [i,k] <- 1/(1+exp(-phi [i,k]))

}}

# linear regression

for(i in 1:N){

for(k in 1:(K-1)){

phi [i,k] <-

# intercepts a and annual trend b (primary parameter of interest)

a [k]+b*YR [i]+

# adjustment for demography

beta.a.g [A.G [ID [i]]]+beta.sex [SEX [ID [i]]] +beta.ethnic [ETHNIC [ID [i]]]+

# person- and set-specific random effects

eta [ID [i]]+zeta [SET [i]]

}}

# intercepts a [] should be ordered

for (i in 1:(K-1)) {

da [i] ~ dunif(0, 20)

}

a [1] <- da [1]

for (i in 2:(K-1)) {

a [i] <- a [i-1] + da [i]

}

# priors

b ~ dnorm(0,1.0E-3)

beta.a.g [1] <- 0

for(ag in 2:4){

beta.a.g [ag] ~ dnorm(0,1.0E-2)

}

beta.sex [1] <- 0

beta.sex [2] ~ dnorm(0,1.0E-2)

beta.ethnic [1] <- 0

for(et in 2:6){

beta.ethnic [et] ~ dnorm(0,1.0E-2)

}

for(j in 1:J){

eta [j] ~ dnorm(0,tau.eta)

}

for(js in 1:S){

zeta [js] ~ dnorm(0,tau.set)

}

tau.eta ~ dgamma(.01,.01)

tau.set ~ dgamma(.01,.01)

# priors for missing ETHNIC

for(j in 1:J){

ETHNIC [j] ~ dcat(p.ethnic [])

A.G [j] ~ dcat(p.a.g [])

SEX [j] ~ dcat(p.sex [])

}

p.ethnic [1:6] ~ ddirch(alpha [1:6])

p.a.g [1:4] ~ ddirch(alpha1 [1:4])

p.sex [1:2] ~ ddirch(alpha2 [1:2])

}
